# Thermo-Mechanical Behavior of Aluminum Matrix Nano-Composite Automobile Disc Brake Rotor Using Finite Element Method

**DOI:** 10.3390/ma15176072

**Published:** 2022-09-01

**Authors:** Palani Sivaprakasam, Esayas Abebe, Robert Čep, Muniyandy Elangovan

**Affiliations:** 1 Department of Mechanical Engineering, Addis Ababa Science and Technology University, Addis Ababa P.O. Box 16417, Ethiopia; 2 Department of Machining, Assembly and Engineering Metrology, Faculty of Mechanical Engineering, VSB-Technical University of Ostrava, 708 00 Ostrava, Czech Republic; 3 Department of R&D, Bond Marine Consultancy, London EC1V 2NX, UK

**Keywords:** aluminum alloy, aluminum matrix nano-composites, disc brake, finite element analysis, thermo-mechanical analysis

## Abstract

Analysis of mechanical and thermal behaviors during braking has become an increasingly important issue in many transport sectors for different modes of transportation. Brake failure generated during braking is a complex phenomenon confronting automobile manufacturers and designers. During braking, kinetic energy is transferred to thermal energy, resulting in the intense heating of disc brake rotors that increases proportionally with vehicle speed, mass, and braking frequency. It is essential to look into and improve strategies to make versatile, thermally resistant, lightweight, high-performance discs. As a result, this study uses the finite element method to conduct a thermo-mechanical analysis of aluminum alloy and aluminum matrix nano-composite disc brake rotors to address the abovementioned issues. The FEA method is used for the thermo-mechanical analysis of AMNCs for vented disc brake rotor during emergency braking at 70 km/h. From the results obtained, aluminum base metal matrix nano-composites have an excellent strength-to-weight ratio when used as disc brake rotor materials, significantly improving the discs’ thermal and mechanical performance. From the result of transient thermal analysis, the maximum value of heat flux obtained for aluminum alloy disc is about 8 W/mm^2^, whereas for AMNCs, the value is increased to 16.28 W/mm^2^. The result from static analysis shows that the maximum deformation observed is 0.19 mm for aluminum alloy disc and 0.05 mm for AMNCs disc. In addition, the maximum von Mises stress value of AMNC disc is about 184 MPa. The maximum von Mises stress value of aluminum alloy disc is about 180 MPa. Therefore, according to the results, the proposed aluminum base metal matrix nano-composites are valid for replacing existing materials for disc brake rotor applications.

## 1. Introduction

Nano-composites are composites with nanometer-scale dimensions in at least one phase. They are made of a matrix material with nanoscale reinforcing components such as particles, whiskers, fibers, nanotubes, etc. Nano-composite materials have materialized as appropriate substitute materials for incredulous restrictions of micro-composites [1]. The increasing demand for high fuel efficiency and low-weight components in automobile industries is challenging for automobile engineers. It has resulted in the development of innovative materials that perform better [2].

Aluminum matrix nano-composites (AMNCs) are a unique class of metal matrix nano-composites that can solve this problem [3]. The excellent thermal and mechanical properties of AMNCs make them the best candidate for manufacturing lightweight automobile parts [4]. In addition, AMNCs have better wear resistance, crack resistance, improved mechanical strength properties, and thermal conductivity compared to cast iron and pure aluminum metal. Due to their excellent properties, they are current and future appropriate materials for various applications in sectors such as the automobile, aerospace, and structural applications [5].

**Figure 1:**
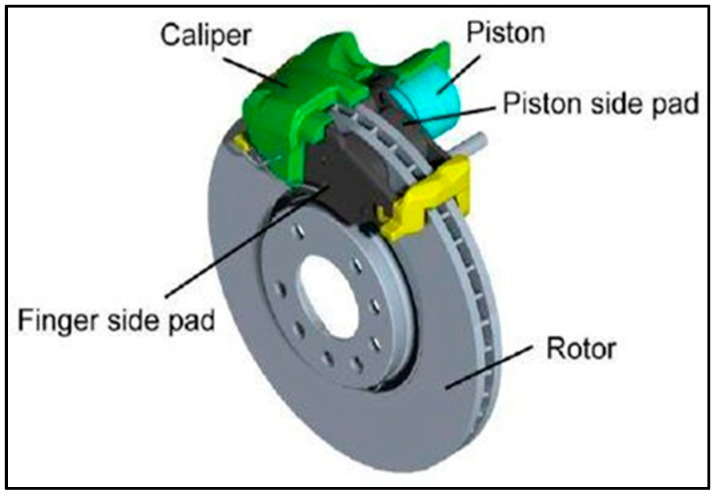
Typical disc brake used in modern vehicles [8].

A brake is a device that applies frictional resistance to a moving element to stop the vehicle or slow it down to a necessary speed from a given initial speed. The brake absorbs the kinetic energy of the moving component while performing this function, and the energy received by brakes is ultimately released in the form of heat [6]. Disc brakes are a form of brake that uses calipers to decelerate a vehicle through friction. Disc brakes are used on the front of all modern vehicles, while some have front and rear disc brakes [7]. Figure 1 shows a typical disc brake used in modern vehicles.

Various materials would be capable of meeting the requirements for a good disc brake performance. Cast iron was frequently used for disc brake rotors due to its superior metallurgical stability, lower cost, and relative simplicity of manufacture. Other materials are employed in high-performance braking applications when the component is exposed to extremely high temperatures. Because of their low density, aluminum alloys containing silicon carbide can also be used [9].

The braking system, a critical system of an automobile, is essential to the vehicle’s safe operation. Automotive braking systems are in charge of reducing vehicle speed, bringing the vehicle to a stop, preventing unwanted acceleration while driving downhill, and keeping the vehicle stationary once it has stopped. This could be accomplished by forcing brake pads against a rotating disc, resulting in frictional forces that slow the rotating disc and, as a result, reduce or stop the car’s speed [10].

Since the late 1930s, researchers have been studying brake failure, and there has been significant progress in reducing noise levels. There are numerous experimental, analytical, and numerical methods for investigating the problem of brake failure. Vibrations caused by friction in vehicle disc brakes are of great interest to the automotive industry. Numerous customer complaints arise from brake failures, which result in high warranty costs. Automobile engineers develop silent brakes using computational and experimental simulations and physical vehicle tests. The importance of computational simulations has increased in the automotive industry due to shorter product development cycles and cost-cutting requirements [11].

The wide range of applications of AMCs in the aerospace, automotive, and other industries is directly related to their appropriate analysis and investigations using experimental or numerical methodologies. Aluminum alloys and silicon carbide-based aluminum metal matrix composites were experimentally studied for friction and wear by Kalyan K S et al. [5]. The test was conducted for sliding speeds of 3.14 m/s and 3.77 m/s and load ranges of 10 N to 30 N in dry and lubricated environments, respectively. Under dry conditions, coefficient of friction of both the matrix alloy and the composite decreases with an increase in load, whereas it increases with an increase in sliding speeds. On the other hand, wear rates of both aluminum matrix alloy and the composites increase with the increase in load and sliding speeds.

Thermal and mechanical properties of silicon carbide (SiC) reinforced in aluminum (Al336) matrix with different weight proportions (2.5%, 5%, 7.5%, 10%, and 12.5% Wt) were studied by corresponding testing methods. The presence of porosity and silicon molecules causes the thermal conductivity of the Al/SiC to rise with increasing percentages of particulate SiC up to 10%. As a result, the feasibility of adopting such an (Al/SiC) metal matrix composite in the automotive brake disc system increases when compared to conventional cast-iron brake discs [12] in terms of the brake disc’s thermal and mechanical performance.

In contrast, Yu [13] used an analytical model to investigate the effects of initial braking velocity (IBV) and Al2O3-SiC (3D)/Al alloy composite thickness. The FEM and computational fluid dynamic (CFD) models of the brake discs’ 3D transient thermo-stress coupling were presented. The results showed that the lower temperature and thermal stress were distributed uniformly on the wear-resistant surface, dominated by the Al alloy brake disc’s high conductivity and cooling ability. Despite this, Nosa et al. [14] investigated a computer model for heat generation and dissipation during disc brake braking with different materials combinations. Modeling and analysis of aluminum matrix nano-composite minimal studies were performed. Hence, there is broad scope for investigating the mechanical and thermal behavior of aluminum matrix nano-composites through the finite element method.

Maheshwari et al. [15] used a multi-criteria decision-making method to design an optimal solid ventilated brake disc. They simultaneously optimized the fatigue life and axial deflection by selecting suitable values for inboard plate thickness, outboard plate thickness, vane height, effective offset, and center hole radius. In related work, Kalita et al. [16] developed a surrogate model optimized by a gray wolf optimizer to maximize the fatigue life while considering the axial deflection as a constraint. They reported an improvement of 21% over the previously published results. Shinde et al. [17] applied several multi-criteria decision-making methods such as a technique for order of preference by similarity to ideal solution (TOPSIS), evaluation based on distance from average solution (EDAS), VlseKriterijumska Optimizacija I Kompromisno Resenje (VIKOR), and multi-objective optimization based on ratio analysis (MOORA) to select the most suitable materials for brake pad lining.

The thermomechanical behavior of the braking system is significantly influenced by temperature. As a result, when there is a heat effect, the brake disc generally deforms and the contact pressure of the pad increases [18]. The temperature increase of the disc and the friction pad interface during braking affects the system’s efficiency. The increase in temperature causes thermal distortion, brake fading, brake fluid vaporization, and brake screaming, among other things [19]. The method of fundamental calculation, which takes into account the essential couplings between the various phenomena, the transitory character of the thermal history of the disc, the behavior elastic of material, the orthoradial thermomechanical gradients, and the rotation of the disc, was implemented for the numerical prediction of the thermomechanical fields which are established in the disc [20]. The study indicated that employing aluminum matrix composites caused the heavy-duty braking process to reach the lowest temperature, while using various types of disc brake material caused the process to get the most significant temperature (cast iron and CCM). Additionally, using carbon-ceramics material, the braking procedure produced the lowest strains [21].

Jiang, L. et al. [22] did a theoretical model of a three-dimensional (3D) transient temperature field for Al alloy brake discs with Al_2_O_3_-SiC (3D) to discuss the effects of initial braking velocity (IBV) and thickness of Al_2_O_3_-SiC (3D)/Al alloy composite wear-resisting layer. The results indicated the maximum friction temperature was not affected by the thickness of the wear-resisting layer. The maximum friction temperature of the brake discs increased with the increase of the IBV. The addition of B_4_C and Gr particles may have given the matrix a more substantial foundation by increasing its wear resistance [23]. The brake pad material operates at a substantially lower temperature because of the superior heat dissipation of the Al/Al_2_O_3_ submicron combination. Brake pad material was found to have a 20% greater coefficient of friction when sliding against gray cast iron up to 2 m/s [24].

The primary research was performed in disc brakes with microparticles reinforced composites materials, but very few studies have been conducted on nanoparticle-reinforced composites. Therefore, this research focuses on finite element analysis on failure analysis of aluminum matrix nano-composite disc brake reinforced with alumina (Al_2_O_3_).

## 2. Methodology and Materials

### 2.1. Materials for Disc Brake Rotor

The brake system should fulfill the following requirements to have a good performance brake system:

- The brakes must be strong enough to stop the car in an emergency within a minimum distance.
- During braking, the driver must maintain good vehicle control to prevent skidding.
- The brakes must have improved anti-fade properties, meaning their efficiency should not deteriorate.
- The brakes must have the maximum thermal resistance.

### 2.2. Matrix Material for Disc Brake Rotor

Aluminum alloys have been more popular recently due to their excellent qualities, including better strength-to-weight ratio, ease of production, great flexibility, good thermal conductivity, and a pleasing surface finish [25,26]. The most widely accessible aluminum alloy is 6061 aluminum to overcome aluminum metal’s drawbacks. It is a wrought aluminum alloy used extensively in the automobile industry [27]. As a result, aluminum 6061 alloy was chosen as the matrix material for this study.

### 2.3. Reinforcement Material for Disc Brake Rotor

Based on the essential requirements of the disc brake, Al_2_O_3_ at the nanoscale was chosen for this investigation. The characteristics of materials become recognizable and predictable at the nanoscale scale. The physical and chemical properties of nano-materials, such as stability, hardness, conductivity, reactivity, optical sensitivity, melting temperature, etc., can be controlled to enhance the general characteristics of conventional materials [9] due to their small dimensions.

Engineering challenges can be solved using the finite element analysis method, which uses numerical simulation to identify solutions to issues that are challenging to solve physically. Rotor disk’s CAD model (SolidWorks 2021) was imported into Ansys Workbench to perform a thermomechanical study. The thermomechanical analysis enables temperature loading in static structural by linking the steady state thermal and static structural module.

## 3. Results and Discussion

### 3.1. Finite Element Modeling of Disc Brake

The FEM is a computational technique for precisely solving mechanical engineering problems. It is a mathematical modeling approach involving the discretization of a continuous domain using building block entities known as finite elements connected by nodes [7,28,29]. Belhocine et al. [30,31] simulated the brake disc’s structural deformation using the FE software ANSYS. It was discovered that the observed induced stress depended on variables including speed, contact pressure, and coefficient of friction. ANSYS 15 was used to conduct the FE analysis and build the CAD model. The Tetrahedron element type, a higher order four-node thermal element, was used for the thermal analysis.

**Figure 2:**
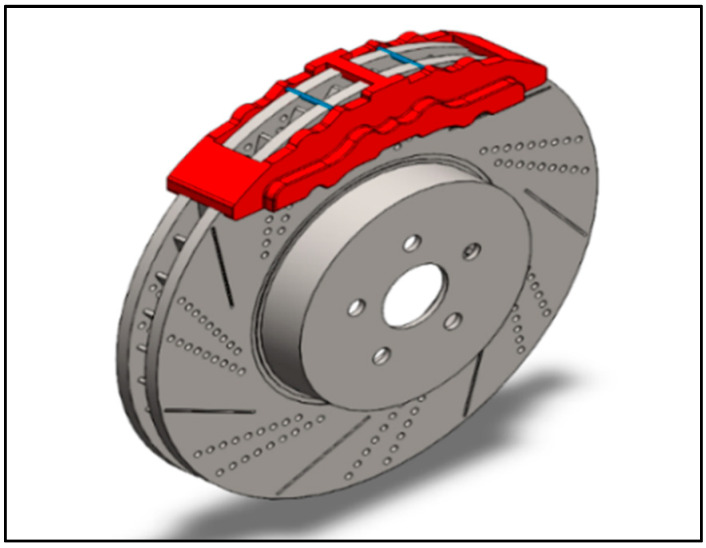
Three-dimensional representation of disc brake rotor.

The geometry of the disc brake rotor is known to influence the rotor’s total exposed area, material composition, and airflow pattern, among other factors. SolidWorks 2021 was used to create this study’s three-dimensional disc brake model. Figure 2 shows the 3D representation of the disc brake rotor. The detailed dimensions of a disc for performing thermal-mechanical calculations are recapitulated in Table 1.

### 3.2. Theoretical Approaches and Calculations of Disc Brake Rotor

The basic assumptions are:

- All kinetic energy at disc brake rotor surface is converted into heat energy.
- Single stop braking scenario where the vehicle is taken.
- Maximum value of pressure developed between disc brake rotor and pad is 1 MPa.
- The disc material is considered homogeneous and isotropic so that Young’s modulus, Poisson’s ratio, and the thermal expansion coefficient remain constant.

#### Braking Energy

During the braking process, the vehicle’s kinetic energy is transformed into heat energy. Therefore, the vehicle has significant masses in rotation. Consequently, the contribution of rotating kinetic energy is considered [30]. The estimation of kinetic energy is calculated using Equations (1)–(7).

(1) $$\mathrm{KE}=\frac{1}{2}\;\times \;m\;\times \;({v_{1}}^{2}-{v_{2}}^{2})+\frac{1}{2}\;\times \;I\;\times \;({\omega_{1}}^{2}-{\omega_{2}}^{2})$$

where,

m—mass of vehicle, kg
v_1_—velocity at the beginning of braking, m/s
v_2_—velocity at the end of braking, m/s
I—mass moment of inertia of rotor, Kg.m^2^
ω_1_—angular velocity of rotor at the beginning of braking, rad/s
ω_2_—angular velocity of rotor at the end of braking, rad/s

Assuming single vehicle stop and when the vehicle stops completely velocity at the end of braking (v_2_) and ending angular velocities will be zero.

v_2_ = ω_2_ = 0 (2)

(3) $$\mathrm{KE}=\frac{1}{2}\;\times \;m\;\times \;v^{2}+\frac{1}{2}\;\times \;I\;\times \;{\omega_{0}}^{2}$$

where:

KE—initial kinetic energy of the vehicle.
m—mass of the vehicle
I—polar inertial moment of rotating parts.

In this case, all the rotating parts are attached to the wheel set’s axle, and the rotation axis can be defined as the tangent line connecting the rail heads’ contact points with the vehicle wheelset. It is the axis of the parallel wheel axles. As a result, the rotating radius equals the brake disc radius (rd2).

(4) $$\mathrm{KE}\;=\;\frac{1}{2}\;\times \;m\;\times \;v^{2}+\frac{1}{2}\;\times \;I\;\times \;{\omega_{0}}^{2}\;=\;\frac{1}{2}\;\times \;m\;\times \;v^{2}\;[1+\frac{I}{mrd2}]$$

The rotating masses are represented by the phrase [$\frac{I}{mrd2}$], which has a value of 0.1 and is used here. Assuming the only passenger inside the car is the driver alone and their mass is 60 kg, and the vehicle is free from any cargo, the total kinetic energy in the vehicle will be:

(5) $$\mathrm{KE}=\frac{1}{2}\left(1.1\right)\;\times \;m\;\times \;v^{2}$$

(6) $$\mathrm{KE}=\frac{1}{2}\left(1.1\right)\;\times \;(3060+60)\;\times \;(41.67{)}^{2}$$

(7) KE=2928.07 KJ

### 3.3. Boundary Conditions

**Figure 3:**
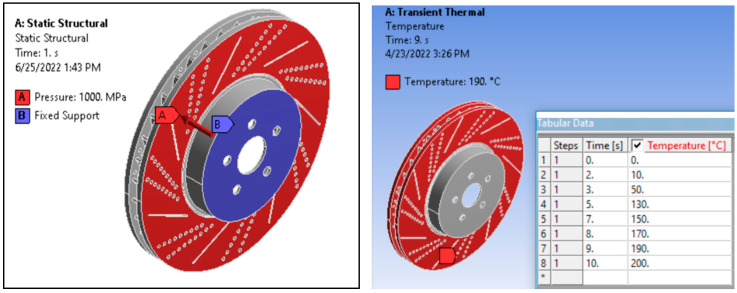
Boundary conditions for mechanical and thermal analysis.

The disc is subjected to 1 MPa pressure force, which is exerted between the disc and brake pad friction surface. The rotating elements in the wheelset, including the disc, rotate at the rotational speed of 120 rad/s. The temperature between the brake pad and the disc surface is assumed to be 200 °C, with a reference temperature of 22 °C for zero-thermal-strain and a convection heat transfer coefficient of 1.24 × 10^−6^ W/mm^2^ °C. The boundary conditions applied for mechanical and thermal analysis are shown in Figure 3.

### 3.4. Static Structural Result

The static structural investigation is applied to a structure when the loads and boundary conditions do not vary over time and remain stationary. It is a crucial tool for determining how components are affected by applied static loading systems and boundary conditions in deformations, stresses, and strains.

#### 3.4.1. Equivalent (Von Mises) Stress

**Figure 4:**
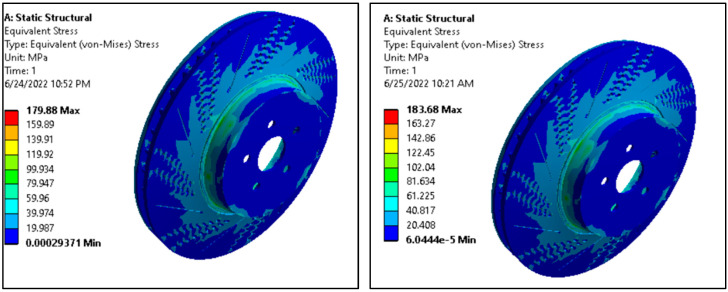
Von Mises stress of aluminum alloy vs. AMNCs ventilated disc.

The equivalent (von Mises) stress values for both aluminum alloy ventilated disc and AMNC ventilated disc is shown in Figure 4.

The highest von Mises stress values of AMNC disc were found to be about 184 MPa, which is drastically less than the maximum permissible stress value, about 845 MPa for AMNC, as shown in Table 2. On the other hand, the maximum Von Mises stress values for aluminum alloy discs were revealed to be about 180 MPa, which is close to the maximum permissible stress value, 310 MPa, for aluminum alloy, as seen in Table 2. As a result, as per von Mises stress failure theory, under the same boundary condition, loading type, and magnitude, AMNC discs show excellent property versus the aluminum alloy disc. Von Mises stress of maraging steel brake disc and radially grooved disc brake were found to be approximately 202 MPa and 137 MPa, respectively [32].

#### 3.4.2. Total Deformation

**Figure 5:**
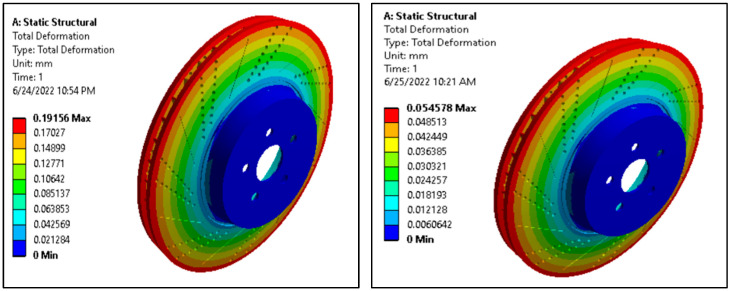
Total deformation of aluminum alloy and AMNCs ventilated disc.

The result of total deformation for the aluminum alloy disc and AMNC disc is presented in Figure 5.

From structural analysis (ANSYS v19.2 workbench software, ANSYS Inc., California, USA), the maximum deformation observed is 0.19 mm for aluminum alloy ventilated disc, and 0.05 mm AMNCs ventilated disc. The maximum deformation of AMNC disc is lower than the maximum deformation of aluminum alloy, indicating that, at the same loading type and magnitude, AMNC disc brake rotors show more excellent resistance to deformation than aluminum alloy discs. The total deformation for rotor in case of stainless steel and gray cast iron is approximately 0.7 mm and 0.8 mm, respectively. From this, it is observed that stainless steel performed better as compared to gray cast iron [33]. It is also observed from literature review [34] that for stainless steel, the total deformation is much less, as compared to gray cast iron. Aluminum alloy experiences less deformation than magnesium alloy, according to past studies. The SiC-reinforced composites have the least distortion compared to the other two models, performing even better [35]. In contrast to [36], from which the disc and pad parameters as well as the material properties were derived, the design strategy of using gradually increasing oval hole areas appears to have been successful [37].

### 3.5. Dynamic/Modal Analysis of Brake Disc

#### 3.5.1. Mode Shape

**Figure 6:**
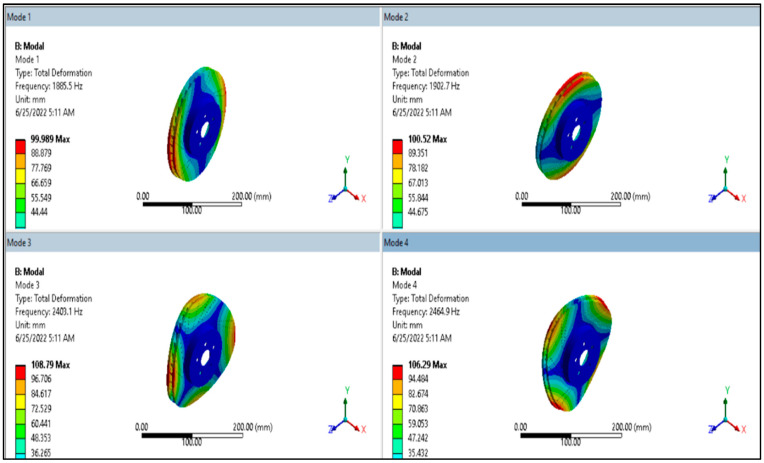
Mode shape result of aluminum alloy disc.

**Figure 7:**
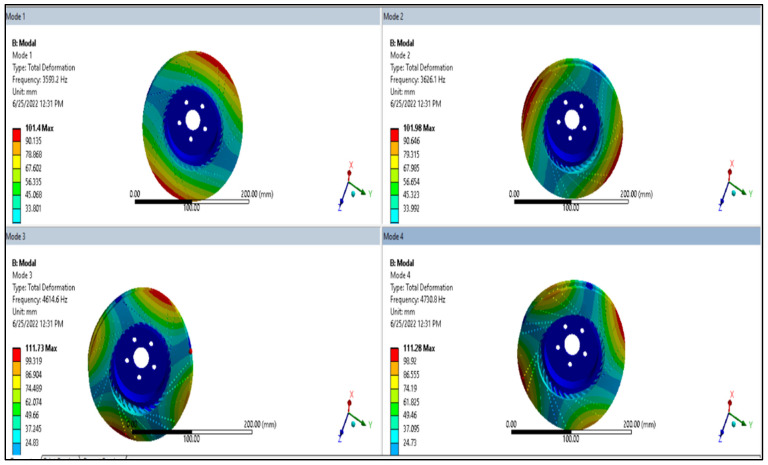
Mode shape result of AMNC disc.

The mode shape result from ANSYS modal analysis is presented in Figure 6 and Figure 7 for aluminum alloy disc and for AMNC disc, respectively.

From Figure 5 and Figure 6, it can be seen that total deformation value is different for each mode. For example, maximum deformation of AMNC at mode 1 is approximately 101 mm, at mode 2 is 102 mm, at mode 3 is 112 mm, and at mode 4 is 111 mm. However, the maximum deformation values for aluminum alloy are about 100 mm at mode 1, 100.5 mm at mode 2, 108.8 mm at mode 3, and 106.3 mm at mode 4. From this result, it is clear to see that AMNCs have higher deformation at each mode than aluminum alloy discs. Under the same loading condition AMNCs show better resistance to deformation than aluminum alloy.

#### 3.5.2. Harmonic Response

**Figure 8:**
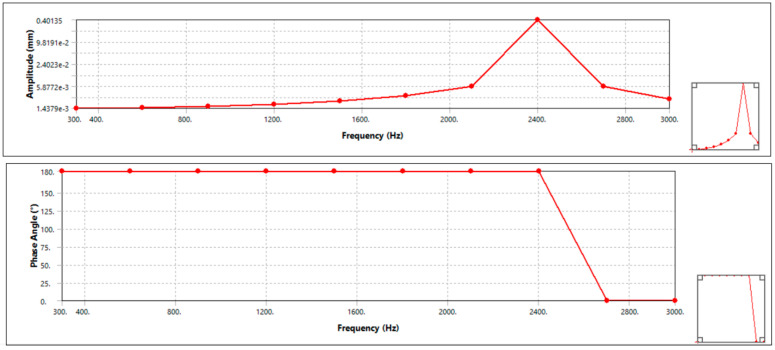
Harmonic response of aluminum alloy disc.

**Figure 9:**
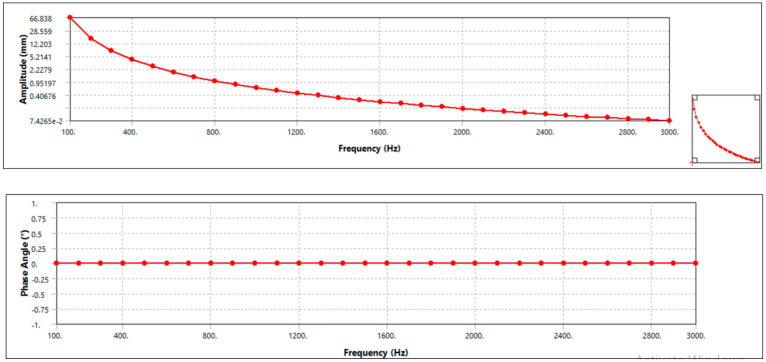
Harmonic response of AMNCs disc.

Meshing is similar to modal since the model in ANSYS project is shared between modal and harmonic, and the boundary condition is also similar with static structural analysis. Only the analysis setting is changed. It is done for the frequency range from 0 Hz up to 3000 Hz. After setting the analysis setup, it is solved for frequency response of deformation. The result is shown in the Figure 8 (aluminum alloy disc brake) and Figure 9 (AMNC disc brakes).

The result shows that the maximum amplitude for aluminum alloy disc, which is around 0.401 mm, occurs at natural frequency value of 2400 Hz, whereas maximum amplitude is less than 0.407 mm when natural frequency is around 2400 Hz for AMNC disc, i.e., maximum amplitude AMNC is less than aluminum alloy disc. From this, it can be seen that the disc made from AMNC has lower vibration, because it has lower amplitude value compared with aluminum alloy-made disc.

### 3.6. Transient Thermal Analysis

Thermal analysis determines the temperature distribution and associated thermal quantities in a system or component in static and dynamic circumstances. Thermal quantities such as temperature distributions, heat loss or gain, and total and directional heat fluxes are all part of a typical thermal study (whether static or transient).

#### 3.6.1. Temperature Distribution

**Figure 10:**
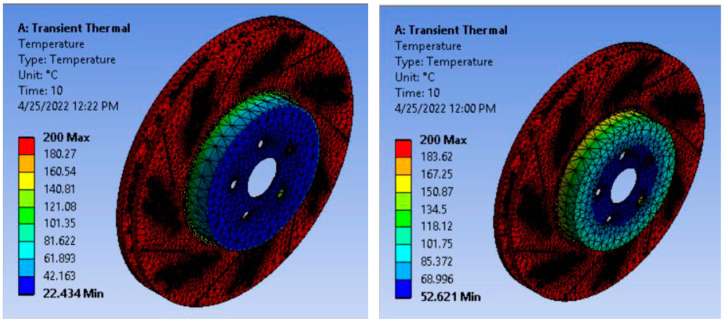
Temperature distribution in aluminum alloy and AMNCs ventilated disc.

Temperature distribution in transient thermal analysis of aluminum alloy and aluminum matrix nano-composite ventilated disc brake rotor was conducted on ANSYS 19.2 workbench. The corresponding result is displayed in Figure 10.

The temperature distribution of aluminum alloy and AMNCs ventilated disc are shown in Table 3. The temperature distribution at 10 s in an aluminum alloy disc increases substantially from about 22 °C to 200 °C, as seen in Figure 10. The temperature distribution for AMNCs is between 52.6 °C and 200 °C. The similar observation found for temperature distributions of different disc brake material are cast iron, stainless steel, and carbon–carbon composites whose value ranges from 25 to 221 °C, 25 to 202.5 °C, and 25 to 91.5 °C, respectively [38]. Hence, the braking condition is too severed, and a high amount of heat is generated between the disc surface and brake pad. AMNCs show better temperature resistance than aluminum alloy discs.

#### 3.6.2. Total Heat Flux

**Figure 11:**
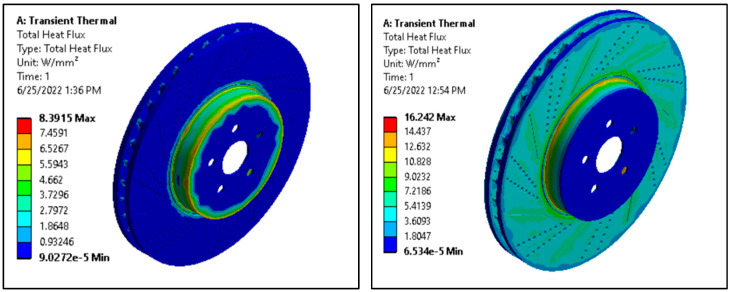
Total heat flux of aluminum alloy and AMNCs disc.

The higher heat flux value indicates higher heat transfer values per unit area. So, the material with a higher heat flux value is to be selected to withstand overheating disc brake rotors. Figure 11 presents the total hat flux result obtained from the ANSYS workbench.

The maximum heat flux value obtained for aluminum alloy disc is about 8.4 W/mm^2^, whereas, for AMNCs, the value is increased to about 16.2 W/mm^2^. Hence, AMNCs discs have a higher value of heat flux than aluminum alloy discs; they provide better cooling and dissipate more heat than aluminum alloy rotors.

### 3.7. Validation of Result

The simulation results of thermo-mechanical analysis of aluminum alloy matrix nano-composite disc brake rotor were compared with the previously reported experimental results conducted design analysis and observed values. The composite materials were chosen as suitable hybrid composite materials based on high yield strength and better deformation resistance properties. The result obtained from this work was validated using appropriate numerical simulation software, ANSYS workbench software package [8].

**Figure 12:**
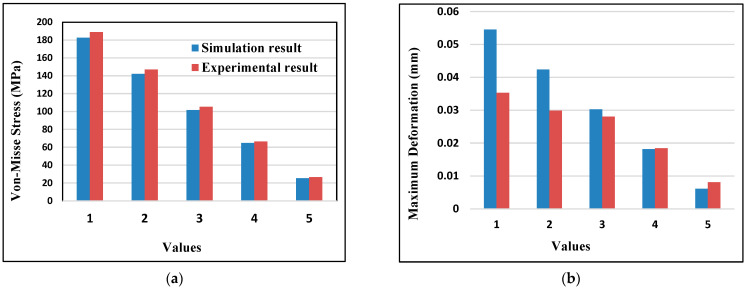
Variation of (**a**) von Mises stress and (**b**) maximum deformation value.

Figure 12 shows the closeness of results between the simulation results of AMNCs discs and the experimental result of Al-SiC/MMC discs for validation purposes. It can be noted that the simulation and experimental findings exhibit nearly the same trend. A slight difference of about 0.0192 mm in maximum deformation was observed, which was caused by a slight variation in loading conditions and the geometry of disc brakes. The simulation result agrees well with the experimental result regarding maximum deformation with a little difference in deformation value. The maximum von Mises stress value of AMNCs discs was about 183 MPa, and the maximum von Mises stress was about 189 MPa. A slight variation, about 6.2 MPa, between the von Mises stress value of AMNCs and von Mises stress of Al-SiC/MMC is revealed, which is also caused by the difference in loading conditions and geometry of disc brakes taken. The average error % values for the von Mises stress in the experimental (Al-SiC/MMC) and simulation data are less than 4%. Moreover, it is observed that deformation values for experimental and simulation results are 8%. Generally, a good agreement between simulation result of AMNCs disc and Al-SiC/MMC disc, even though some difference is observed. The aluminum metal matrix composite will produce the desired results for disc brake rotors with cross-drilled vents. It can withstand thermal stresses by friction between brake pads and disc rotor surfaces while dissipating heat more quickly. Due to its low deformation, high strain and stress levels, and high heat flow compared to other materials, aluminum metal matrix composite material is recommended [39].

## 4. Conclusions

Thermo-mechanical analysis of AMNCs for ventilated disc brake rotor during emergency braking at 70 km/h is carried out using the FEA method.

The static analysis results show that the maximum deformation observed is about 0.19 mm for aluminum alloy disc and about 0.06 mm for AMNCs disc. In addition, the maximum von Mises stress value of the AMNC disc is about 184 MPa. The maximum von Mises stress value of aluminum alloy discs is about 180 MPa using the same boundary conditions; AMNC discs exhibit superior strength to aluminum alloy discs.

The transient thermal analysis shows that the maximum heat flux value obtained for aluminum alloy disc is about 8 W/mm^2^, whereas, for AMNCs, the value is increased to about 16 W/mm^2^. Therefore, the aluminum matrix nano-composite rotor provides better cooling and dissipates more heat as compared to aluminum alloy rotors.

From dynamic/modal analysis, the total deformation value of AMNCs discs was lower than that of aluminum alloy discs. The maximum deformation of AMNCs disc was between approximately 101 mm and 111 mm. The maximum deformation values for aluminum alloy discs were between about 100 mm and 106 mm.

In addition, the harmonic response result revealed that the maximum amplitude for aluminum alloy disc, around 0.401 mm, occurs at a natural frequency value of 2464.9 Hz. The maximum amplitude is less than 0.406 mm when the natural frequency is around 2021.6 Hz for AMNC disc, i.e., the maximum amplitude AMNC is less than the aluminum alloy disc. From this, it can be concluded that AMNC discs have lower vibration than aluminum alloy discs.

From both static and dynamic analysis, it can be seen that AMNCs have higher mechanical properties than aluminum alloy disc brake rotors under the same boundary conditions and loading system.

AMNCs disc brake rotors have a higher strength to weight ratio and higher thermal resistance under severe braking conditions than aluminum alloy disc brake rotors. Therefore, replacing existing conventional aluminum alloy disc brake material with AMNC material is possible with better thermal resistivity, good performance, and strength-weight ratio.

## Figures and Tables

**Table 1:** Dimensions of Toyota Land Cruiser HZJ76R hard top [9].

Brake Rotor Parameter Name	Parameter Value (Units)
The outer diameter of the disc rotor (D_o_)	0.302 (m)
The inner diameter of the disc rotor (D_i_)	0.100 (m)
Number of holes	6
Thickness of disc brake rotors	0.048 (m)
Drilled hole diameter	0.010 (m)
Top speed (V)	52.78 (m/s)
Wheel base	0.2730 (m)
Wheel diameter	0.68(m)

**Table 2:** Result of von Mises stress of aluminum alloy and aluminum alloy nano-composite ventilated disk.

Materials	Von Mises Stress (MPa)	Allowable Stress (MPa)
Aluminum alloy disc	179.88	310
Aluminum matrix nano-composite disc	183.68	845.21

**Table 3:** Temperature distribution in aluminum alloy and AMNCs ventilated disc.

Time (s)	Aluminum Alloy Disk		AMNCs Disk	
	Minimum (°C)	Maximum (°C)	Minimum (°C)	Maximum (°C)
1.	5	22	5	21.92
2.	9.98	21.99	9.55	21.71
3.	20.82	50	20.78	50
4.	21.85	90	21.68	90
5.	21.97	130	22.90	130
6.	21.99	140	25.62	140
7.	22.01	150	30.05	150
8.	22.08	170	36.12	170
9.	22.20	190	43.70	190
10.	22.43	200	52.62	200

## Data Availability

The data presented in this study are available in the article.
